# 

**Published:** 1911-05

**Authors:** 


**A Manual of Physical Diagnosis.** By **Brefney Rolph O'Reilly**, M.D., C.M. (F.T.M.C. Toronto, M.R.C.S. England, L.R.C.P. London), Demonstrator in Clinical Medicine and in Pathology, University of Toronto; Assistant Physician to St. Michael's Hospital, Toronto; Physician to Toronto Hospital for Incurables. 12mo, pp. 390. With six plates and forty-nine other illustrations. Philadelphia: P. Blakiston's Son & Company, 1012 Walnut Street. 1911.

A great deal of information is compressed in this attractive little volume on the handling and physical examination of patients. Following a short introduction, which contains some excellent hints as to management, the book covers in orderly sequence very fully the field of physical diagnosis. An appendix is added which deals with clinical pathology. The chapters on examination of lungs and heart are particularly good and up-to-date.

The book is one which can be heartily recommended to both students and practitioners. A. E. W.

